# Wellbeing and chronic lung disease incidence: The Survey of Health, Ageing and Retirement in Europe

**DOI:** 10.1371/journal.pone.0181320

**Published:** 2017-07-20

**Authors:** Judith A. Okely, Seif O. Shaheen, Alexander Weiss, Catharine R. Gale

**Affiliations:** 1 Centre for Cognitive Ageing and Cognitive Epidemiology, Department of Psychology, University of Edinburgh, Edinburgh, United Kingdom; 2 Centre for Primary Care and Public Health, Barts and The London School of Medicine and Dentistry, Queen Mary University of London, London, United Kingdom; 3 Department of Psychology, University of Edinburgh, Edinburgh, United Kingdom; 4 MRC Lifecourse Epidemiology Unit, University of Southampton, Southampton, United Kingdom; Universite de Bretagne Occidentale, FRANCE

## Abstract

**Background:**

Previous studies indicate that psychosocial factors can impact COPD prevalence. However, research into this association has predominantly focused on negative factors such as depression. The aim of this study was to examine whether high subjective wellbeing is associated with a lower risk of developing COPD.

**Methods:**

The sample consisted of 12,246 participants aged ≥50 years from the Survey of Health, Ageing and Retirement in Europe. We used Cox proportional hazards regression to examine the relationship between wellbeing (measured using the CASP-12) and incidence of COPD over a follow-up period of 9 years.

**Results:**

There was a significant association between wellbeing and COPD risk. In age-adjusted analyses, a standard deviation increase in CASP-12 score was associated with a reduced risk of COPD; hazard ratios (95% confidence intervals) for men and women were 0.67 (0.60–0.75) and 0.80 (0.73–0.87) respectively. After additional adjustment for demographic and health behaviour variables, this association remained significant for men but not for women: the fully-adjusted hazard ratios were 0.80 (0.70–0.91) and 0.91 (0.82–1.03) respectively.

**Conclusions:**

Greater wellbeing is associated with a reduced risk of COPD, particularly in men. Future research is needed to establish whether gender reliably moderates this association.

## Introduction

Chronic obstructive pulmonary disease (COPD) is a leading cause of morbidity and mortality worldwide [1]. This progressive disease is characterised by persistent airflow limitation caused by a combination of small airways disease (obstructive bronchiolitis) and emphysema [2]. Established risk factors for COPD include smoking, exposure to air pollutants, chronic lung infections, older age and genetic factors [1]. Recent reports suggest that prevalence rates of COPD may have stabilised in some developed countries as a result of reduced smoking prevalence [1]. Given this recent decline in tobacco use, Rosenberg et al. [1] suggest that future trends in the prevalence of COPD will be driven by factors other than smoking prevalence.

In addition to traditional risk factors, there is evidence that psychosocial factors impact on COPD prevalence. Current evidence for this association comes predominantly from studies into negative psychosocial factors such as depression or anxiety. For instance, a longitudinal study involving 14,682 men and women, found that major depression at baseline was associated with a significantly higher risk of developing COPD over a two year follow up period. The effect size was not substantially changed by the inclusion of gender, age, health care use and smoking [3]. A second study examined the association between psychological distress and risk of COPD and found that psychological distress was associated with an increased risk over the 3 year follow up period in women (n = 2203) but not in men (n = 1682) [4].

There are a number or mechanisms that could account for the link between psychosocial factors and COPD risk. Depression or anxiety may confer an increased risk due to associations with traditional risk factors including higher levels of smoking and physical inactivity [4]. In addition, negative psychosocial factors could impact COPD risk via more direct physiological pathways. Previous studies have documented a link between depression or anxiety and elevated levels of inflammatory markers [5,6] as well as chronic inflammation and the development and progression of COPD [7]. Currently, research into the association between wellbeing and COPD risk is limited. However, positive states such as high wellbeing have been linked with health protective behaviours (including physical activity) and lower levels of inflammatory biomarkers. Thus, high wellbeing may be associated with a reduced COPD risk.

An examination of both positive and negative factors in relation to COPD risk is warranted as previous studies into wellbeing and health indicate that wellbeing can impact on health independently of negative affect [8,9]. One previous study has examined the association between subjective wellbeing (as measured by the CASP-19) and the risk of COPD (defined as a diagnosis of chronic bronchitis or emphysema) [10]. The study sample consisted of people aged 50 and over from the English Longitudinal Study of Ageing (ELSA). In people below the age of 65, higher wellbeing was associated with a reduced risk of developing COPD over an 8 year follow up period. This effect remained significant after adjusting for health behaviours, depressive symptoms, demographic variables, BMI and history of asthma. This finding indicates that high wellbeing may be protective against the risk of COPD; and that this effect is in part independent of negative emotion.

The association between wellbeing and COPD risk may have clinical relevance; if high wellbeing confers a reduced risk of COPD, interventions that increase wellbeing may help in reducing COPD prevalence. Previous studies have illustrated that public interventions can effectively increase wellbeing among older adults [11–13]. However, before such an approach can be recommended, further research is needed to confirm whether high wellbeing reliably reduces COPD risk, and to identify potential mechanisms that underlie this association. The aim of the current study was to address these two issues. We assessed the association between wellbeing and COPD risk in a nationally representative European sample of community-dwelling individuals aged 50 years and over. This data set allowed us to control for a range of covariates in our analysis including health behaviours and depressive symptoms.

## Methods

### Study population

The Survey of Health, Ageing and Retirement in Europe (SHARE) is a multi-national prospective cohort study of people aged 50 and over [14,15]. Based on probability samples, SHARE is designed to be representative of the older community-dwelling population in 11 European countries (Denmark, Sweden, Austria, France, Germany, Switzerland, Belgium, the Netherlands, Spain, Italy and Greece) and Israel. Sampling techniques varied by country (depending on what data was available) and included simple random sampling from national population registers, multi-stage sampling using regional/local population registers and single or multi-stage sampling using telephone directories followed by screening in the field. People were excluded from the study if they were born after 1954, or if they were incarcerated, hospitalized or out of the country during the entire survey period, unable to speak the country’s language(s) or had moved to an unknown address. Eligible individuals were recruited by telephone or in person at their home address. Participants have been interviewed biennially since 2004. Data from waves 1–5 (2004–2013) were used in the current analysis. SHARE has been reviewed and approved by the Ethics Committee of the University of Mannheim [16] and all participants provided written consent.

### Wellbeing

Wellbeing at wave 1 was assessed with the CASP-12, participants respond to 12 questions on a four point Likert scale. Scores range from 0–48 with higher scores indicating higher wellbeing. The CASP-12 is an abridged version of the CASP-19 [17] and was developed for use in the SHARE sample. The CASP-12 has been validated in an older Spanish community dwelling sample and has satisfactory internal consistency [18]For the study sample, CASP-12 scores were relatively stable over the follow up period. The test re-test correlation coefficient for CASP-12 scores at wave 1 and wave 5 was high: *r* = 0.52, *p* = <0.001.

### COPD

At each wave (excluding wave 3), participants were asked whether a doctor had ever told them that they had “COPD such as chronic bronchitis or emphysema.” Participants that did not report a diagnosis at wave 1 but reported a diagnosis in a subsequent wave were classified as incident cases. Data on date of COPD diagnosis was not available, for the purposes of the analysis, the month and year of the interview at which the participant first reported a diagnosis of COPD was used as the date of diagnosis.

### Covariate variables

We chose covariates variables that have been linked with wellbeing and COPD in previous studies. These included age, gender and height, depressive symptoms, socioeconomic status, chronic physical conditions, health behaviours and body mass index. All previously associated with wellbeing [19–25] and with COPD risk [1,3,26–30]. The EURO-D was used to assess symptoms of depression [31]. The scale consists of 12 items–all of which are taken from the Geriatric Mental State [32]. The EURO-D is internally consistent and has two factors: ‘affective suffering’ and ‘motivation’ [31,33]. Additional variables adjusted for were SES and education. Socioeconomic status was indexed by total household assets, gross value of home, value of any other real estate, value of any share of business and value of any vehicles minus mortgage of main residence. The sample was divided into quintiles according to total household wealth. Level of education was classified using the International Standard Classification of Education (ISCED-97). Education categories were: pre-primary or primary, lower secondary, upper or post-secondary and first or second stage tertiary. We adjusted for physical activity, alcohol consumption, smoking status, pack years and BMI. Participants reported how often they engaged in vigorous and or moderate physical activity. Response options were: ‘more than once a week’, ‘once a week’ and ‘one to three times a month’, and ‘hardly ever or never’. As has been done previously [34], we used these responses to create three categories: physical inactivity, moderate but not vigorous activity at least once a week and vigorous physical activity at least once a week. Participants reported frequency of alcohol consumption, response options were ‘almost every day’, ‘5 or 6 days a week’, ‘3 or 4 days a week’, ‘once or twice a week’, ‘once or twice a month’, ‘less than once a month’ and ‘not at all in the last 6 months’. As previously [34], we used these responses to create 4 categories: ‘more than once a week’, ‘once a week’, ‘one to three times a month’ and ‘hardly ever or never’. Participants reported their smoking status (non-smoker, former smoker or current smoker). Former and current smokers reported the number of cigarettes, cigars or pipes they smoked per day and the number of years they had smoked. For the pack years variable, number of cigars and pipes per day were converted to the equivalent number of cigarettes (1 cigar or pipe = 2.5 cigarettes). We then derived the number of cigarette packs smoked per day (n of cigarettes per day/20) and multiplied the number of cigarette packs per day by the number of years smoked. The pack years variable was coded as 0 for non-smokers. BMI (kg/m^2^) was derived from participant self-reported height and weight. Participants were categorised according to World Health Organisation guidelines as being underweight (below 18.5 kg/m^2^), normal weight (18.5–24.9 kg/m^2^), overweight (25–29.9 kg/m^2^), and obese (30 kg/m^2^ or above) [35]. Finally, adjustments were made for prevalent hypertension, high cholesterol, diabetes, heart attack, stroke, cataracts, osteoporosis, arthritis and asthma at wave 1. These chronic conditions commonly co-occur with COPD [36] and have been associated with lower wellbeing [37,38].

### Analytical sample

At wave 1, 30, 816 participants took part. Our sample included 12,246 of these participants. Participants were excluded if they reported a diagnosis of COPD at wave 1 or did not know whether they had been diagnosed with COPD or if they refused to respond to the question (n = 1,664; 5%). We also excluded participants that only participated at wave 1 (n = 7,363; 25%), had missing wellbeing data (n = 7970; 37%) or had missing covariate data (n = 1573; 11%).

Participants with missing wellbeing data were older, more likely to be female, consumed less alcohol, were less physically active, were less likely to smoke, had fewer years of education and were more likely to report a history or stroke, diabetes and heart attack. These participants also had a higher depression score and had lower SES.

### Statistical analysis

Proportional hazards regression was conducted to examine the association between CASP-12 scores at baseline and incidence of COPD over the follow-up period. Survival time was calculated from the date of the wave 1 interview to the date of COPD diagnosis, or the date of the last follow up interview. The mean duration of follow up was 6 years. Preliminary analysis (predicting COPD risk) indicated a significant interaction between gender and CASP-12 (*p =* 0.02). Consequently, proportional hazards regression analysis was conducted for women and men separately.

There were 8 different adjustment models, the first model adjusted for age and the subsequent 6 models adjusted for age and each covariate in turn (this approach allowed us to identify the amount of variance explained by each covariate); the final model adjusted for age and all covariates variables (height, SES, depressive symptoms, comorbidities, health behaviours and BMI).

To rule out the effect of reverse causality (i.e. undiagnosed pre-existing lung disease influencing wellbeing), the regression was repeated excluding the first two years of follow up. Hazard ratios (HR) and 95% confidence intervals (CI) are expressed according to a standard deviation (SD) increase in CASP-12 score.

To test for possible bias due to missing data, multiple multivariate imputation was used to impute values of covariates with missing values. This approach relies on the assumption that data are missing at random—meaning that the pattern of missingness is systematic and can be predicted by observed data [39]. We assumed data were missing at random as missingness was significantly correlated with other measured variables [39]. Missing data were imputed for the sample of participants that took part at wave 1, did not report a diagnosis of COPD at wave 1 and had information on incident COPD (n = 21,789). The imputation models included survival time, COPD incidence and the covariates; 35 imputed datasets were generated using chained equations imputation.

## Results

Table 1 shows the baseline characteristics of the sample (n *=* 12,246) according to tertiles of wellbeing. Mean wellbeing scores for the lowest, middle and highest tertiles were 30.67 (SD = 3.92), 38.06 (SD = 1.41) and 43.48 (SD = 1.94) respectively. On average, participants with higher wellbeing were younger, taller, wealthier, more educated, more likely to be male, less likely to report a history of chronic disease excluding asthma and less likely to smoke. They also had lower depressive symptom scores, lower BMI, were more physically active and consumed more alcohol.

**Table 1:** Baseline characteristics stratified according to tertiles of CASP-12 scores (lowest, middle and highest subjective wellbeing) Total N = 12,246.

Characteristics	Lowest	Middle	Highest	*p-*trend^a^
Age (yrs), *M* (SD)	64.81 (10.44)	63.07 (9.77)	62.09 (8.96)	<0.001
EURO-D score *Mdn* (IQR)	3 (1–5)	2 (1–3)	1 (0–2)	<0.001
Total wealth (€) *M* (SD)	335,408.52 (134,8262.59)	589,167.40 (220,7638.04)	853,964.48 (261,0017.07)	<0.001
Female, No. (%)	2281 (58.64)	2093 (54.05)	2379 (53.06)	<0.001
BMI (kg/m^2^) *M* (SD)	26.93 (4.46)	26.36 (4.19)	25.96 (3.98)	<0.001
Height (cm)	166.45 (8.93)	168.51 (8.91)	169.49 (8.84)	<0.001
Physical Activity, No. (%)				<0.001
Physically inactive	679 (17.46)	309 (7.98)	242 (5.40)	
Moderate physical activity	1500 (38.56)	1453 (37.53)	1422 (31.71)	
Vigorous physical activity	1711 (43.98)	2110 (54.49)	2820 (62.89)	
Alcohol consumption, No. (%)				<0.001
More than once a week	916 (23.55)	968 (25.00)	1247 (27.81)	
Once a week	804 (20.67)	1156 (29.86)	1574 (35.10)	
One to three times a month	811 (20.85)	879 (22.70)	870 (19.40)	
Hardly ever or never	1359 (34.94)	869 (22.44)	793 (17.69)	
Smoking status, No. (%)				<0.001
Non-smoker	768 (19.74)	757 (19.55)	846 (18.87)	
Former smoker	945 (24.29)	1140 (29.44)	1414 (31.53)	
Smoker	2177 (55.96)	1975 (51.01)	2224 (49.60)	
Pack Years *Mdn* (IQR)	25.00 (10.00–42.00)	20.00 (7.50 37.50)	18.50 (7.00–35.00)	<0.001
Education, No. (%)				<0.001
Pre-primary or primary	1718 (44.16)	1067 (27.56)	829 (18.49)	
Lower secondary,	716 (8.41)	696 (17.96)	844 (18.82)	
Upper or post-secondary	1005 (25.84)	1269 (32.77)	1555 (34.68)	
First or second stage tertiary,	451 (11.59)	840 (21.69)	1256 (28.01)	
History of heart attack, No. (%)	556 (14.29)	429 (11.08)	321 (7.16)	<0.001
History of high cholesterol, No. (%)	876 (22.52)	813 (20.99)	807 (17.99)	<0.001
History of stroke, No. (%)	179 (4.60)	115 (2.97)	87 (1.94)	<0.001
History of asthma, No. (%)	144 (3.72)	147 (3.80)	140 (3.12)	0.19
History of arthritis, No. (%)	991 (25.48)	648 (16.74)	490 (10.93)	<0.001
History of osteoporosis, No. (%)	421 (10.82)	264 (6.82)	184 (4.10)	<0.001
History of cataracts, No. (%)	338 (8.69)	253 (6.53)	244 (5.44)	<0.001
History of diabetes, No. (%)	421 (10.82)	264 (6.82)	184 (4.10)	<0.001
History of hypertension, No. (%)	338 (8.69)	253 (6.53)	244 (5.44)	<0.001

^a^ statistical significance is based *χ*^2^ tests or one-way ANOVA, as appropriate.

Table 2 displays the HRs for incident COPD for men and women according to a SD increase in CASP-12 score. There were 715 incident cases reported between waves 2 and 5 (Table 2). People with higher CASP-12 scores had a significantly lower risk of incident lung disease after adjusting for age. This reduction appeared to be greater for men than for women. A SD increase in CASP-12 score was associated with a 33% (HR: 0.67; 95% CI: 0.60–0.75) decrease in COPD risk in men and a 20% (HR: 0.80; 95% CI: 0.73–0.87) decrease in COPD risk in women. This association remained significant but was attenuated in men and women after adjusting for each covariate separately. Adjusting for depressive symptoms and SES led to the highest percentages of attenuation, 25 or 15% and 20 or 15%, respectively. Adjusting for BMI and history of relevant chronic conditions led to a higher percentage of attenuation for women than for men.

**Table 2:** Hazard ratios (95% confidence intervals) for incident COPD in women and men according to a SD increase in CASP-12 score.

Covariates Adjusted for	Gender	HR (95% CI)	% Attenuation
Age	Women	0.80 (0.73–0.87)**	
	Men	0.67 (0.60–0.75)**	
Age+ Wealth + Education	Women	0.85 (0.77–0.93)*	25%
	Men	0.72 (0.64–0.80)**	15%
Age+ Height	Women	0.79 (0.72–0.87)**	-5%
	Men	0.69 (0.62–0.76)**	6%
Age+ EURO-D	Women	0.84 (0.76–0.94)*	20%
	Men	0.72 (0.64–0.82)**	15%
Age + Comorbidities	Women	0.85 (0.77–0.93)*	25%
	Men	0.70 (0.63–0.78)**	9%
Age+ Health Behaviours	Women	0.83 (0.76–0.92)**	15%
	Men	0.70 (0.64–0.79)**	9%
Age+ BMI	Women	0.83 (0.75–0.91)**	15%
	Men	0.68 (0.61–0.76)**	3%
All Covariates	Women	0.91 (0.82–1.03)	55%
	Men	0.80 (0.70–0.91)*	39%

** *p* <0.001

* *p* <0.05

In the model adjusted for all covariates, the association between higher wellbeing and lower COPD risk remained significant for men (HR: 0.80; 95% CI: 0.70–0.91) but was no longer significant for women (HR: 0.91; 95% CI: 0.82–1.03). In the fully adjusted model, factors significantly associated with increased COPD risk included older age, being female, smoking, being underweight, having fewer years of education, history of arthritis and history of asthma.

The observed association between CASP-12 score and COPD risk was little changed by the exclusion of cases diagnosed in the first two years of follow-up or the exclusion of participants that reported a history of asthma at wave 1. The pooled effect sizes from analysis with imputed information were similar to those obtained from analysis employing the sample with complete data–suggesting that missing covariate data did not significantly bias the results. See S1 Table for a comparison of results.

## Discussion

This study successfully replicated the finding that wellbeing is protective against COPD risk [10]. The association between higher CASP-12 scores remained significant following additional adjustment for covariate variables (wealth, education, height, depressive symptoms, comorbidities, health behaviours and BMI) in men but not in women.

Adjusting for traditional risk factors attenuated the association between wellbeing and COPD risk. SES and depressive symptoms emerged as significant covariates. This effect is unsurprising, SES is associated with levels of wellbeing [23] and risk of COPD [27]. Depressive symptoms are strongly associated with lower wellbeing [19]; in the SHARE sample, there was a significant negative correlation between the two measures (*r =* 0.64 *p <* 0.001 after adjusting for unreliability). Depression has also previously been identified as a risk factor for COPD [26]. Adjusting for BMI and health behaviours including alcohol consumption, smoking status, pack years and physical activity attenuated the association between wellbeing and COPD further. Again, this finding is as expected considering the documented association between wellbeing and health behaviours [21] and health behaviours (smoking in particular) and risk of COPD [1]. Health behaviours may partially mediate the association between wellbeing and COPD risk. However, it is also possible that these factors are confounders of this risk association; that is, health behaviours and BMI may impact on levels of wellbeing and the risk of COPD. Longitudinal mediation analysis could help establish the role of health behaviours in the association between wellbeing and COPD risk.

We observed a stronger association between wellbeing and COPD risk in men compared with women. There is no clear consensus regarding gender as a modifier of effects of psychosocial risk factors. It is plausible that the gender interaction observed in this study is specific to the association between wellbeing and COPD. There are a number of possible explanations for this effect. For instance, Watson et al. [40] suggest that women experience a more severe form of COPD than men. It is possible that wellbeing is not potentially protective against this more severe form of the disease. Alternatively, COPD is underdiagnosed in women as it is traditionally considered a ‘male’ disease (mainly due to previously higher smoking rates among men) [41]. It is possible that less reliable diagnosis of COPD in women resulted in an underestimation of the association between wellbeing and COPD for women in this study.

For the men in the sample, established risk factors and comorbidities did not fully account for the association between wellbeing and the risk of COPD. This suggests that additional mechanisms may account for the association. One possibility is that wellbeing impacts directly on physiological processes relevant to COPD risk. This causal pathway has been proposed by Kubzansky et al. [42]. The development and progression of COPD is associated with an abnormal inflammatory response. Wellbeing could impact this process as previous studies have documented a significant association between positive affect and biomarkers of inflammation [43,44]. The data in SHARE did not allow for an assessment of this causal pathway. However, further research testing whether inflammatory processes mediate the association between wellbeing and COPD risk is warranted.

There are a number of additional factors (not recorded at wave 1 of SHARE) that may underlie the association between wellbeing COPD risk. Firstly, diet quality may play a role. Poor diet quality (high intake of processed meats, refined grains and sugar sweetened drinks) has been identified as a risk factor for COPD [45] and has also been associated with low wellbeing [21]. A further possibility is that the association between wellbeing and COPD risk is driven by the link between wellbeing and the likelihood of reporting disease symptoms. There is evidence that people with high positive affect report fewer and less severe disease symptoms than people with low positive affect even when objective markers of disease are held constant [49]. It is plausible that participants with high wellbeing were less likely to receive a formal diagnosis as they reported fewer disease symptoms. Finally, it is also possible that wellbeing and COPD are not causally related. For instance, work environment may act as a third variable confound. Factory and construction work is associated with an increased risk of COPD due to occupational exposures to vapours, gas, dust and fumes [46]. Working in a loud or noxious environment is also associated with reduced wellbeing [47,48]. Thus, our results could, at least in part, reflect the effect of work environment on COPD risk and levels of wellbeing. Early life or genetic factors could also confound the association between wellbeing and COPD risk. The development of lung function in infancy has been identified as a significant predictor of pulmonary health in old age [50]. Risk factors for abnormal lung development include premature birth or low birthweight, tobacco exposure during and after pregnancy and childhood respiratory illness [50]. Stafford et al. [51] report that childhood illness and family psychosocial factors are related to wellbeing in early old age. The contribution of genetics to the association between wellbeing and COPD risk remains to be explored.

Strengths of the study include the sample–which was large and representative of people age ≥ 50 living in Europe and Israel. The available data allowed for adjustment for many covariate variables. Our study also had some limitations. Firstly, over a third of our participants (37%) were excluded due to missing wellbeing data. Participants with missing wellbeing data differed from those included in the sample on several key variables, including depressive symptoms and health behaviour. Excluding these participants may therefore have biased the results; however, analysis with imputed missing covariate data yielded similar effect sizes to those obtained for the sample with complete data suggesting that this exclusion did not result in any bias. Secondly, diagnosis of COPD was assessed using a self-report measure—which has been associated with some degree of reporting bias [52]. Although, others have found that self-report measures of physician diagnosed conditions provide a valid estimate of disease prevalence [53]. Finally, although depressive symptoms were controlled for, it was not possible to control for two psychosocial factors previously associated with pulmonary health: perceived stress and hostility [54,55] as well as several established risk factors that may be related to wellbeing, including diet, exposure to air pollutants and early life influences.

Our results support the idea that greater wellbeing is associated with a reduced risk of COPD. In addition to conferring the inherent benefits of increased wellbeing [11–13], interventions designed to improve wellbeing may help in reducing the prevalence of COPD among older adults. Additional research is needed to further delineate the causal pathways between wellbeing and COPD risk. Factors including inflammatory processes, symptom severity or symptom reporting, diet, work environment, early life exposures as well as perceived stress or hostility in adulthood may be implicated in this association.

## Supporting information

S1 Table Hazard ratios (95% confidence intervals) from analysis with imputed missing covariates and from analysis with complete data. Model 1: Adjusted for age. Model 2: Further adjusted for total net wealth, education, comorbidities, depressive symptoms, smoking, alcohol intake, physical activity and BMI. ** p <0.001 * p <0.05
(DOCX)
