# Supplementary material for: Wellbeing and chronic lung disease incidence: The Survey of Health, Ageing and Retirement in Europe
Source: PLoS One. 2017 Jul 20;12(7):e0181320. doi: 10.1371/journal.pone.0181320 (PMC5519137; doi:10.1371/journal.pone.0181320)
Supplement: S1 Table — Model 1: Adjusted for age. Model 2: Further adjusted for total net wealth, education, comorbidities, depressive symptoms, smoking, alcohol intake, physical activity and BMI. ** p <0.001 * p <0.05 (DOCX) [file pone.0181320.s001.docx]

S1 Table

*Hazard ratios (95% confidence intervals) from analysis with imputed missing covariates and from analysis with complete data*

| **Gender** | **Analysis** | **Model 1**  **HR(95%-CI)** | **Model 2**  **HR(95%-CI)** |
| --- | --- | --- | --- |
| Women | Imputed | 0.78 (0.72-0.84)** | 0.90 (0.81-0.99)* |
|  | Complete | 0.80 (0.73-0.87)** | 0.91 (0.82-1.03) |
| Men | Imputed | 0.67 (0.60-0.74)** | 0.82 (0.74-0.92)* |
|  | Complete | 0.67 (0.60-0.75)** | 0.80 (0.70-0.91)* |

Model 1: Adjusted for age. Model 2: Further adjusted for total net wealth, education, comorbidities, depressive symptoms, smoking, alcohol intake, physical activity and BMI.

** p <0.001 * p <0.05
